# Different Fatty Acid Supplementation in Low-Protein Diets Regulate Nutrient Utilization and Lipid and Amino Acid Metabolism in Weaned Pigs Model

**DOI:** 10.3390/ijms24108501

**Published:** 2023-05-09

**Authors:** Qingsong Tang, Wenxue Li, Zhongxiang Ren, Qi Ding, Xie Peng, Zhiru Tang, Jiaman Pang, Yetong Xu, Zhihong Sun

**Affiliations:** Laboratory for Bio-Feed and Molecular Nutrition, College of Animal Science and Technology, Southwest University, Chongqing 400715, China

**Keywords:** low-protein diet, fatty acid, nutrient metabolism, weaned pigs

## Abstract

This study was conducted to evaluate the effects of a low-protein (LP) diet supplemented with sodium butyrate (SB), medium-chain fatty acids (MCFAs) and n-3 polyunsaturated fatty acids (PUFAs) on nutrient utilization and lipid and amino acid metabolism in weaned pigs. A total of 120 Duroc × Landrace × Yorkshire pigs (initial body weight: 7.93 ± 0.65 kg) were randomly assigned to five dietary treatments, including the control diet (CON), LP diet, LP + 0.2% SB diet (LP + SB), LP + 0.2% MCFA diet (LP + MCFA) and LP + 0.2% n-3 PUFA diet (LP + PUFA). The results show that the LP + MCFA diet increased (*p* < 0.05) the digestibility of dry matter and total P in pigs compared with the CON and LP diets. In the liver of the pigs, the metabolites involved in sugar metabolism and oxidative phosphorylation significantly changed with the LP diet compared with the CON diet. Compared with the LP diet, the altered metabolites in the liver of the pigs fed with the LP + SB diet were mainly associated with sugar metabolism and pyrimidine metabolism; the altered metabolites in the liver of pigs fed with the LP + MCFA and LP + PUFA diets were mainly associated with lipid metabolism and amino acid metabolism. In addition, the LP + PUFA diet increased (*p* < 0.05) the concentration of glutamate dehydrogenase in the liver of pigs compared with the LP diet. Furthermore, the LP + MCFA and LP + PUFA diets increased (*p* < 0.05) the mRNA abundance of sterol regulatory element-binding protein 1 and acetyl-CoA carboxylase in the liver compared with the CON diet. The LP + PUFA diet increased (*p* < 0.05) mRNA abundances of fatty acid synthase in the liver compared with the CON and LP diets. Collectively, the LP diet supplemented with MCFAs improved nutrient digestibility, and the LP diet supplemented with MCFAs and n-3 PUFAs promoted lipid and amino acid metabolisms.

## 1. Introduction

The shortage of high-quality protein sources and the pollution of the environment by excreta are worldwide problems. Low-protein (LP) diets have been extensively researched to address this problem. In recent years, studies have shown that LP diets are beneficial for reducing the use of protein feedstuff and nitrogen emissions [1]. In addition, reducing dietary protein levels during the weaning transition period can reduce excess protein entering the hindgut to ferment and causes worsening of diarrhea [2,3]. However, the adaptation of organisms to a protein-deficient metabolism results in decreased amino acid metabolism, which limits body protein synthesis and growth of weaned pigs [4]. Previous studies have reported that a balanced amino acid composition does not alleviate LP diets-induced reduced amino acid metabolism and growth in weaned pigs [5]. These critical issues need to be addressed before LP diets are widely used in pig production.

Fatty acids serve as important metabolic regulators due to the fact of their roles as energy substrates. The composition and molecular structure of dietary fatty acids, including chain length and number of double bonds, influence the digestion, absorption, and metabolism of nutrients [6]. Fatty acids can be classified based on the length of the hydrocarbon chain into three categories: short-chain fatty acids (SCFAs, with fewer than 6 carbon atoms), medium-chain fatty acids (MCFAs, with 6 to 12 carbon atoms), and long-chain fatty acids (LCFAs, with more than 12 carbon atoms) [7]. Butyrate, an SCFA, was first identified as a major energy source for the intestines and indirectly affects cholesterol synthesis and regulates insulin and glucagon secretion [8]. Previous studies have shown that butyrate is also widely involved in the regulation of tissue metabolic homeostasis and improved feed efficiency in piglets [9,10,11,12]. MCFAs are absorbed directly into the portal blood and may provide immediate energy to enterocytes, facilitating the transition of piglets during the weaning period [6]. A previous study reported that feeding with MCFAs improved nutrient absorption and lipid metabolism in weaned pigs [13]. n-3 Polyunsaturated fatty acids (n-3 PUFAs) are an LCFA and can improve metabolic homeostasis in piglets by affecting insulin and glucagon receptors and ion channels, as well as regulating glucose, total protein (TP) and lipids levels in the liver and plasma [14,15]. To date, there have been few studies on the comparative effects of sodium butyrate (SB), MCFAs and n-3 PUFAs on the nutrient utilization and lipid and amino acid metabolism in weaned pigs.

Given that fatty acids serve as energy substrates and metabolic regulators, we hypothesized that the addition of SB, MCFAs and n-3 PUFAs to the LP diets of weaned pigs can increase the oxidative metabolism of lipids and inhibit the role of amino acids as metabolic fuels, thereby promoting amino acids’ metabolism and deposition as body proteins. Therefore, the purpose of this study was to investigate the comparative effects of an LP diet supplemented with SB, MCFAs and n-3 PUFAs on the nutrient utilization and lipid and amino acid metabolism in weaned pigs. These results may provide new insight into the exploration of the optimal type of fatty acid supplementation in LP diets in weaned pigs.

## 2. Results

### 2.1. Relative Weight of Organ and Nutrient Digestibility

The effects of an LP diet supplemented with fatty acids on the organ weights of the pigs are shown in Table 1. There was no significant difference in the weights of the liver, heart, kidney, pancreas, and spleen of the weaned pigs among the five groups (*p* > 0.05). However, there was an increase (*p* < 0.05) in the relative weight (% of BW) of the liver and heart of pigs in the LP, LP + SB, LP + MCFA and LP + PUFA groups compared with those of pigs in the CON group. Meanwhile, there was an increase (*p* < 0.05) in the relative weight of the pancreas of the pigs in the LP and LP + MCFA groups compared with those of pigs in the CON and LP + SB groups. As shown in Table 2, the dry matter digestibility of pigs fed the LP + MCFA diet was higher (*p* < 0.05) than the CON and LP diets. The total P digestibility of pigs fed the LP + MCFA diet was higher (*p* < 0.05) than the CON, LP and LP + PUFA diets.

### 2.2. Plasma Biochemical Parameters

Plasma TC levels were increased (*p* < 0.05) in pigs fed the LP diet compared with the CON, LP + SB, LP + MCFA, and LP + PUFA diets (Table 3). With regard to hormone levels, there was an increase (*p* < 0.05) in the CCK levels in pigs fed the LP + PUFA diets compared with the LP, LP + SB and LP + PUFA diets (Table 4).

### 2.3. Untargeted Metabolomics

**Figure 1:**
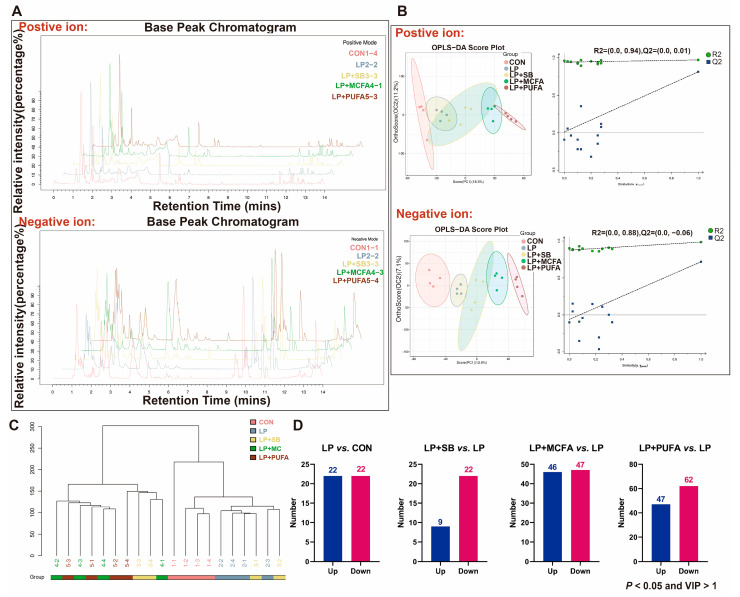
LP diet supplemented with SB, MCFAs and n-3 PUFAs affected the liver metabolome in weaned pigs: (**A**) map of base peak chromatogram from quality control (QC) samples in the positive ion mode and negative ion mode; (**B**) orthogonal partial least squares discriminant analysis (OPLS-DA) score plot and OPLS-DA replacement inspection chart; (**C**) hierarchical clustering tree diagram of population samples, where the clustering method is average-linkage; (**D**) number of differentially metabolites (*p* < 0.05 and VIP > 1) of LP vs. CON, LP + SB vs. LP, LP + MCFA vs. LP and LP + PUFA vs. LP. Four replicates/treatment (*n* = 4), one pig/replicate.

Nontargeted metabolomics were used to investigate the effects of SB, MCFAs and n-3 PUFAs on the hepatic metabolism of the weaned pigs. The map of the base peak chromatogram in the positive ion mode and negative ion mode showed a relatively similar trend among the groups (Figure 1A), and the OPLS-DA score plot and OPLS-DA replacement inspection chart in the positive ion mode and negative ion mode showed good reproducibility and reliable results for each group (Figure 1B). Additionally, hepatic metabolites in the pigs fed the CON diet were similar to those in the pigs fed the LP diet, while the pigs fed the LP + MCFA diet were similar to those in the pigs fed the LP + PUFA diet (Figure 1C).

**Figure 2:**
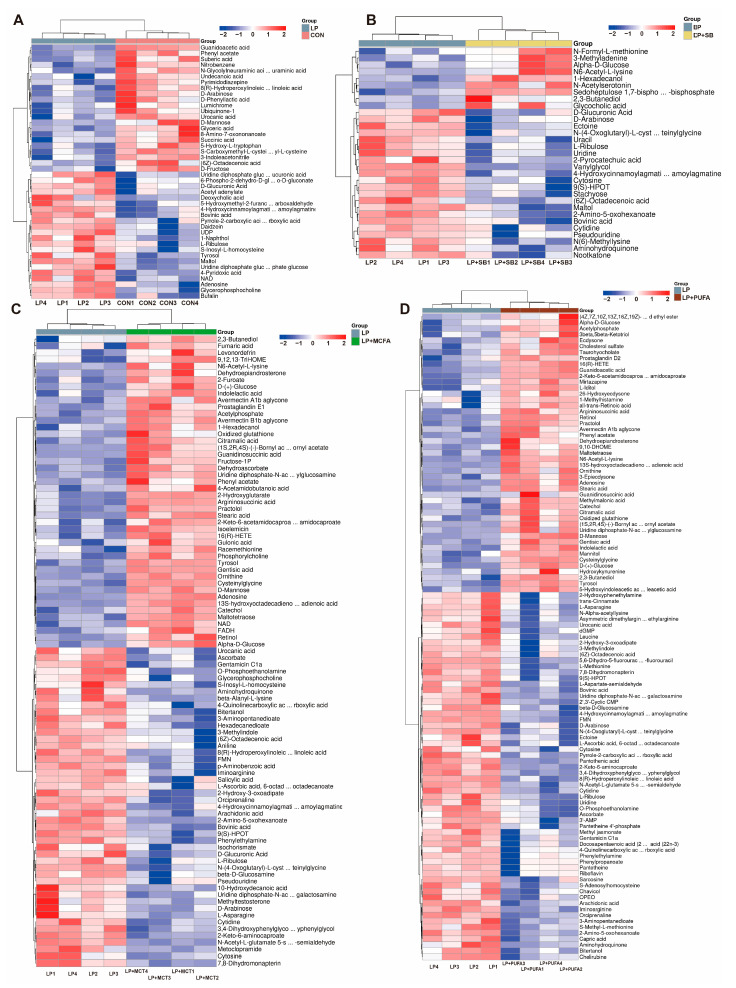
Comparison among groups of differential metabolites in the liver of weaned pigs. Heatmaps demonstrating significantly different metabolites from (**A**) LP vs. CON; (**B**) LP + SB vs. LP; (**C**) LP + MCFA vs. LP; (**D**) LP + PUFA vs. LP. Red indicates high abundance, and blue indicates low abundance. Four replicates/treatment (*n* = 4), one pig/replicate.

**Figure 3:**
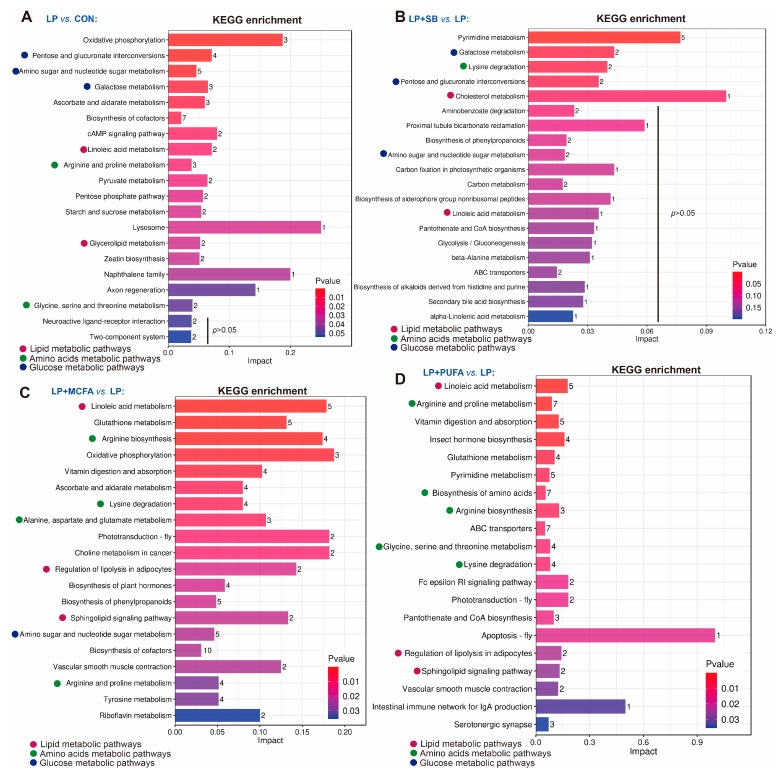
KEGG enrichment pathway analysis of differential metabolites in the liver of weaned pigs: (**A**) LP vs. CON; (**B**) LP + SB vs. LP; (**C**) LP + MCFA vs. LP; (**D**) LP + PUFA vs. LP. Numbers represent the number of differential metabolites; 4 replicates/treatment (*n* = 4), 1 pig/replicate.

A total of 557 significant variations in metabolites were identified with the criteria of an OPLS-DA model VIP > 1 and *p* < 0.05. A total of 44 (22 up and 22 down), 31 (9 up and 22 down), 93 (46 up and 47 down) and 109 (47 up and 62 down) metabolites significantly changed in the four pairwise comparisons (LP vs. CON, LP + SB vs. LP, LP + MCFA vs. LP and LP + PUFA vs. LP) (Figure 1D). The heatmap shows the differentially metabolites in the pairwise comparisons: LP vs. CON (Figure 2A), LP + SB vs. LP (Figure 2B), LP + MCFA vs. LP (Figure 2C) and LP + PUFA vs. LP (Figure 2D). A Kyoto Encyclopedia of Genes and Genomes (KEGG) pathway analysis revealed that the altered metabolites in the LP group were mainly involved in glucose metabolism and ATP production-related pathways, especially pentose and glucuronate interconversions, amino sugar and nucleotide sugar metabolism, galactose metabolism, oxidative phosphorylation, and biosynthesis of cofactors compared with the CON group (Figure 3A). The altered metabolites in the LP + SB group were mainly enriched in galactose metabolism, pentose and glucuronate interconversions, and pyrimidine metabolism (Figure 3B). The altered metabolites in the LP + MCFA and LP + PUFA groups were mainly linked with lipid metabolism and amino metabolism, especially linoleic acid metabolism, regulation of lipolysis in adipocytes, sphingolipid signaling pathway, arginine biosynthesis, lysine degradation, alanine, aspartate and glutamate metabolism, arginine and proline metabolism, and biosynthesis of amino acids (Figure 3C,D).

### 2.4. Metabolic Pathways

**Figure 4:**
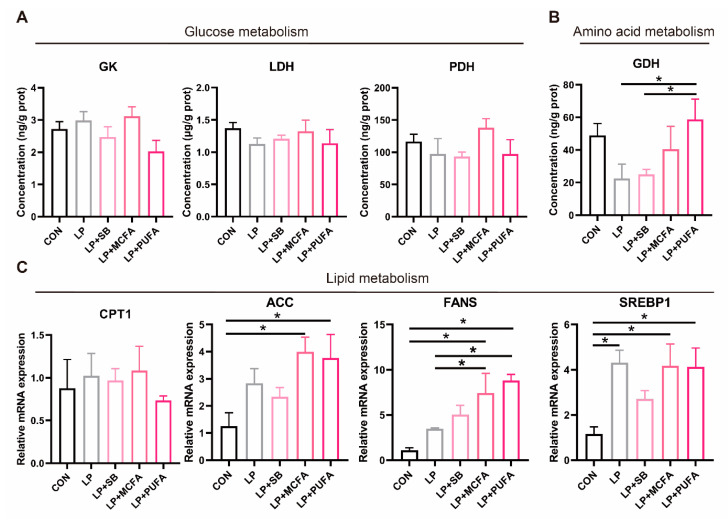
Levels of critical enzymes and transcription factors of glucose metabolism, amino acid metabolism and lipid metabolism pathways: (**A**) concentration of glucokinase (GK), lactate dehydrogenase (LDH), pyruvate dehydrogenase (PDH) and (**B**) glutamate dehydrogenase (GDH) in the liver determined using ELISA; (**C**) real-time quantitative PCR was performed to determine the relative mRNA expression levels of acetyl-CoA carboxylase (*ACC*), fatty acid synthase (*FASN*), carnitine palmitoyl transferase 1 (*CPT1*) and sterol regulatory element-binding protein 1 (*SREBP1*) in the liver. All data are expressed as the mean ± SEM (*n* = 6). * *p* < 0.05; 6 replicates/treatment (*n* = 6), 1 pig/replicate.

The concentration of GDH in the liver of pigs fed the LP + PUFA diet increased (*p* < 0.05) compared with pigs fed the LP diet (Figure 4B). However, there was no significant difference in the concentrations of GK, LDH and PDH in the liver of weaned pigs among the five groups (*p* > 0.05) (Figure 4A). In addition, the expression of acetyl-CoA carboxylase (*ACC*) in the liver increased (*p* > 0.05) in pigs fed the LP + MCFA and LP + PUFA diets compared with pigs fed the CON diet (Figure 4C). The expression of fatty acid synthase (*FASN*) in the liver increased (*p* > 0.05) in pigs fed the LP + MCFA and LP + PUFA diets compared with pigs fed the CON and LP diets. The LP, LP + MCFA and LP + PUFA diets increased (*p* < 0.05) the relative mRNA expression of sterol regulatory element-binding protein 1(*SREBP1*) in the liver of weaned pigs compared with the CON diet.

## 3. Discussion

**Figure 5:**
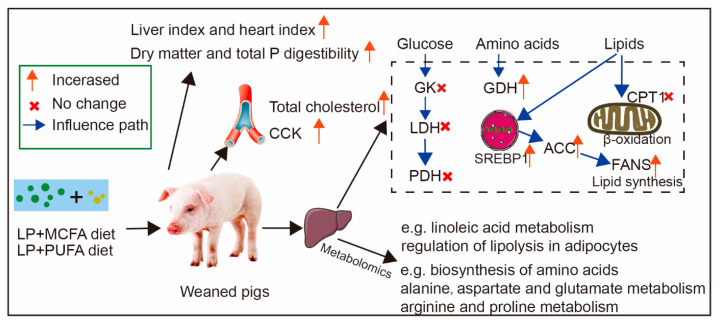
Proposed functional model of fatty acid supplementation in a low-protein diet. The red arrows indicate an increase in the value of the detected parameter compared to CON or LP, and the red crosses indicate no change.

Fatty acid metabolism is known to improve metabolic homeostasis by enhancing lipid synthesis, storage and catabolism in animals [16]. In the present study, LP diets supplemented with fatty acids improved organ development and the digestibility of dry matter and total P in weaned pigs, while it reduced the concentration of TC in the plasma of weaned pigs. The LP + PUFA diet also promoted the secretion of CCK. Moreover, the LP + MCFA diet mainly altered the lipid metabolism and amino metabolism, similar to the changes of the LP + PUFA diet (Figure 5).

The relative weight of organ is a classical parameter for measuring growth development and immune function [17]. In this study, we observed that the LP diet and fatty acid supplementation increased the relative weight of liver and heart of weaned pigs, while both the LP diet and the LP + MCFA diet increased the relative weight (% of BW) of pancreas of weaned pigs. The liver and heart are important metabolic organs and generally reflect the metabolic status of the animals [18,19]. The pancreas regulates nutrient metabolism by secreting hormones, such as insulin, glucagon and GH, and secretes digestive enzymes to facilitate the digestion of fats, proteins and carbohydrates [20]. Previous studies have reported that a 3% reduction in the dietary protein level significantly increased the relative weight of liver of weaned pigs [21]. The results suggested that in order to adapt to lower dietary protein levels, the animals increased their liver and relative weight of heart to achieve metabolic homeostasis; however, fatty acids supplementation did not break this metabolic pattern. Moreover, pigs fed the LP + MCFA diet had higher digestibility of dry matter and total *p* than pigs fed the CON diet, which is consistent with other studies [22]. The improved apparent digestibility of nutrients in pigs by MCFAs may be related to their beneficial effects in increasing gastrointestinal digestive enzyme activity and improving intestinal health [23,24].

Blood biochemical parameters reflect nutrient metabolism, in particular the concentrations of TP, PUN, glucose, TC, TG, LDL-C and HDL-C, while hormone levels, such as insulin and CCK, reflect the amino acid, glucose and lipid metabolisms [25]. In the present study, the LP diet and the fatty-acid-supplemented diet had no significant effect on the concentrations of TP, PUN, glucose, TG, LDL-C and HDL-C in the plasma of weaned pigs. However, there was an increase in the plasma TC in pigs fed the LP diet, whereas the fatty acid supplementation reversed the increase in TC, which in agreement with other studies [26]. The findings suggest that the reduced dietary protein levels in the weaned pigs might impede cholesterol metabolism, while fatty acids supplementation might promote cholesterol metabolism. Cholesterol is mostly broken down and metabolized into bile acids in animals, and bile acids can improve the digestion and absorption of nutrients [27]. We speculated that fatty acid supplementation could facilitate the conversion of cholesterol into bile acids and enhance nutrient digestion and absorption in weaned pigs. Thyroid hormone signaling regulates important biological functions, including the energy expenditure, development and growth of animals [28]. GH, insulin and GLP-1 play an important role in the regulation of glucose metabolism, and their secretion is inhibited by SS [29]. In the present study, T3, T4, insulin, GLP-1, GHRP, GH, SS and leptin were not significantly altered by the experimental diets, indicating that neither dietary protein level reduction nor fatty acid supplementation caused the metabolic disorders. CCK acts as an endocrine satiety signal in response to the digestive state of carbohydrates, lipids and proteins [30]. In addition, CCK could improve nutrient digestion by stimulating gastric acid and digestive enzymes secretion and slowing gastric emptying [31]. In the present study, the plasma concentration of CCK was higher in the pigs fed the LP + PUFA diet than the LP, LP + SB and LP + MCFA diets, suggesting that n-3 PUFAs stimulate CCK secretion. Overall, the LP diet supplemented with SB, MCFAs and n-3 PUFAs reduced the cholesterol level in plasma, and n-3 PUFAs increased nutrition digestibility of the pigs by promoting CCK secretion.

The amino acid, glucose and lipid metabolisms in the liver are dynamic processes [32]. Therefore, we focused on the hepatic metabolism of weaned pigs fed with the LP diets with or without fatty acid supplementation. We found that the LP diet significantly altered 44 metabolites in the liver, wherein KEGG analysis showed that differential metabolites were mainly enriched in pentose and glucuronate interconversions, amino sugar and nucleotide sugar metabolism, galactose metabolism, oxidative phosphorylation and biosynthesis of cofactors. The results suggest that reducing dietary protein levels altered glucose metabolism and ATP production-related pathways. Of note, the levels of nicotinamide adenine dinucleotide (NAD^+^), succinic acid, ubiquinone-1, UDP, uridine diphosphate glucose, uridine diphosphate glucuronic acid and D-glucuronic acid significantly increased in the oxidative phosphorylation pathway and biosynthesis of cofactors pathways. In particular, NAD^+^ is an important coenzyme cofactor in redox reactions and is involved in energy metabolism through oxidative phosphorylation, glycolysis, acetyl-CoA production and the tricarboxylic acid cycle [33,34]. These results suggest that the LP diet promoted energy metabolism by increasing NAD^+^ production and was primarily associated with glucose metabolism and oxidative phosphorylation pathways. We also compared the changes in liver metabolites between the fatty acid supplemented diets and the LP diet in weaned pigs. This study showed that the LP + SB diet significantly altered 31 metabolites in the liver of pigs, which were mainly enriched in the galactose metabolism, pentose and glucuronate interconversions, and pyrimidine metabolism. Moreover, the LP + MCFA and LP + PUFA diets had similar effects on liver metabolites in pigs, altering 93 and 109 metabolites, and both were mainly enriched in the lipid metabolism and amino metabolism, especially linoleic acid metabolism, regulation of lipolysis in adipocytes, sphingolipid signaling pathway, arginine biosynthesis, lysine degradation, alanine, aspartate and glutamate metabolism, arginine and proline metabolism, and biosynthesis of amino acids. Previous studies have reported that MCFA and n-3 PUFA diets can significantly alter the fatty acid composition of animal plasma and tissues [35,36]. These results suggest that the LP + MCFA diet or LP + n-3 PUFA diet had a greater effect on liver metabolism than the LP + SB diet in pigs, mainly altering lipid metabolism and amino acid metabolism. In this study, both the LP + MCFA and LP + PUFA diets significantly increased the lipid levels of 13S-hydroxyoctadecadienoic acid but significantly reduced liver arachidonic acid, bovinic acid (a conjugated linoleic acid), 8(R)-hydroperoxylinoleic acid and O-phosphoethanolamine. Dietary linoleic acid and alpha-linolenic acid are converted into arachidonic acid and eicosapentaenoic acid in pigs [7]. These results suggest that MCFAs and n-3 PUFAs promote the conversion of linoleic acid into other metabolites. Previous studies have reported that LP diets reduce serum-free amino acid concentrations in pigs, usually resulting in impaired muscle protein synthesis and growth [37,38]. In general, animals adapt to a metabolism of chronic protein deficiency by reducing the rate of protein synthesis [39]. In this study, the LP diet supplemented with MCFAs and n-3 PUFAs promoted amino acid synthesis and metabolic pathways, which may compensate for the lack of protein synthesis in the LP diet.

The possible regulatory pathways associated with glucose metabolism, amino acid metabolism and lipid metabolism were investigated using the levels of GK, LDH, PDH and GDH in the liver and the relative mRNA abundance of *ACC*, *FASN*, carnitine palmitoyl transferase 1 (*CPT1*) and *SREBP1* in the liver of weaned pigs. As is known, GK, LDH and PDH are important regulatory enzymes in the process of glycolysis and acetyl-CoA production pathway, reflecting the rate of glucose metabolism [40]. In the present study, fatty acid supplementation in the LP diet had no significant effect on the concentrations of GK, LDH and PDH in the liver of weaned pigs. This confirmed the metabonomic results, and the effects of the different experimental diets on glucose metabolism were relatively small. GDH is one of the main catalytic enzymes of amino acid metabolism and is mainly involved in catalyzing the reversible reaction of oxidative deamination of glutamate into α-ketoglutarate [41]. In this experiment, the concentration of GDH in the liver of pigs fed the LP + PUFA diet increased compared with pigs fed the LP diet. The involvement of GDH in the oxidative deamination process is also a major pathway of nitrogen metabolism in animals [42]. This suggests that the LP diet supplemented with n-3 PUFAs increased the capacity of amino acid metabolism in the liver of weaned pigs, which is beneficial for maintaining energy homeostasis and nitrogen balance.

However, the results of this study show that the LP diet supplemented with fatty acids had no effect on the relative expression of *CPT1*. *CPT1* has been identified as the gatekeeper enzyme for fatty acid entry into mitochondria, controlling the flux of fatty acids through the esterification and oxidation pathways [43,44]. This suggests that LP diet supplementation with fatty acids that promote the lipid metabolism, especially the LP + MCFA diet and LP + PUFA diets, does not cause an increase in fatty acid oxidation and may be used more for fat synthesis. Fat synthesis is mainly influenced by the activity of various key enzymes, including those involved in lipogenesis and lipolysis [45]. Among these enzymes, *ACC* and *FASN* are involved in the ab initio synthesis of fatty acids and could alter the rate of fatty acid biosynthesis and catabolism [46]. The present study showed that the mRNA levels for lipogenesis genes (*ACC* and *FASN*) increased in pigs fed both the LP + MCFA and LP + PUFA diets. *SREBP1*, an important nuclear transcription factor, is indispensable in coordinating the expression of lipogenesis-related genes to promote lipid homeostasis [47]. Indeed, along with elevated levels of adipogenic gene mRNA, we noted that the LP diet supplemented with MCFAs and n-3 PUFAs induced an upregulation of lipid transcription factor (*SREBP1*), which may contribute to sustained adipogenesis and deposition. Overall, an LP diet supplemented with MCFAs and n-3 PUFAs improved amino acid metabolic homeostasis in weaned pigs and promoted lipid metabolism and fat synthesis.

In summary, an LP + MCFA diet can improve nutrient digestibility in weaned pigs. Furthermore, the LP + MCFA diet or LP + n-3 PUFA diet had a greater effect on liver metabolism in pigs than the LP + SB diet, mainly altering lipid and amino acid metabolism. The addition of MCFAs or n-3 PUFAs to LP diets is more beneficial in improving the metabolic homeostasis of weaned pigs. However, further studies are needed to explore the underlying mechanism of the regulation of fat and protein deposition by MCFAs and n-3 PUFAs.

## 4. Materials and Methods

### 4.1. Dietary Treatments and Animal Management

A total of 120 crossbred weaned pigs (Duroc × Landrace × Yorkshire) with an initial body weight of 7.93 ± 0.65 kg (28 day old) were randomly allocated into 5 dietary treatments. Each treatment consisted of six replicate pens, and there were four weaned pigs per pen, balanced for sex. The five experimental diets were formulated based on corn-soybean meal: (1) control diet (CON) containing 20.4% crude protein (CP); (2) LP diet containing 16.5% CP; (3) LP diet supplemented with 0.2% SB (LP + SB); (4) LP diet supplemented with 0.2% MCFAs (LP + MCFA); (5) the LP diet supplemented with 0.2% n-3 PUFAs (LP + PUFA). The SB (content ≥ 98%), MCFAs (content ≥ 50%) and n-3 PUFAs (content ≥ 50%) were obtained from Longyan Xinao Biotechnology Co., Ltd. (Longyan, China). All diets were supplemented with limiting amino acids, including lysine, methionine, tryptophan and threonine, to meet the nutrient requirements for pigs according to the National Research Council 2012 (NRC 2012) [48], and the composition and nutrient profiles are shown in Table 5. The animal experiment lasted for 28 days, and the pigs were transferred to one of five experimental diets over 3 days to gradually reach 100% of the experimental diets on day 4. The pigs were fed the experimental diets three times a day (at 7:30, 12:30 and 17:30) and had ad libitum access to feed and water.

### 4.2. Sample Collection

A total of 300 g feed per experimental diet were collected and stored at −20 °C until further analysis. From d 26 to 28, fresh fecal samples (approximately 200 g per replicate pen per collection) of the pigs were collected and mixed with 10 mL of 10% sulfuric acid per 100 g of feces, and they were stored at −20 °C until further analysis. At the end of the experiment, one pig from each replicate was randomly selected for anterior vena cava blood sampling. The plasma samples were collected using 5 mL anticoagulation tubes containing 100 IU of sodium heparin. The plasma was centrifuged for 10 min at 4 °C and 3000× *g* rpm, and the plasma was separated and stored at −80 °C. After blood collection, thirty pigs (one per replicate) were euthanized by the intravenous injection of sodium pentobarbital solution (40 mg/kg body weight). The pigs were necropsied; the liver, heart, kidneys, pancreas and spleen were separated and weighed; and the relative weight (% of BW) of the organs was calculated. The liver samples were collected and immediately frozen in liquid nitrogen and stored at −80 °C for subsequent analysis.

### 4.3. Analytical Methods

#### 4.3.1. Apparent Total Tract Digestibility

The fecal samples from each pig were thawed and followed with drying at 65 °C for 72 h. Whereafter, all samples of feces and feeds were ground to pass through a 1 mm screen before chemical analysis. Briefly, the dry matter was measured by drying the samples of feces and feeds at 105 °C for 6 h according to the Association of Official Analytical Chemists (AOAC, method 930.15). The CP, Ca, P and ash insoluble in hydrochloric acid (AIA) in the diets and feces were analyzed according to GB/T 6432-2018, GB/T 6436-2018, GB/T 6437-2018 and GB/T 23742-2009, respectively. The indigestible marker AIA was used as an endogenous indicator, and the apparent total tract digestibility of the nutrients was calculated using the following equation: apparent total tract digestibility (%) = 1 − (nutrient content in feces × AIA content in diets)/(nutrient content in diets × AIA content in feces).

#### 4.3.2. Biochemical Parameters

The contents of plasma TP, glucose, total cholesterol (TC), triglycerides (TG), urea nitrogen (PUN), low-density lipoprotein cholesterol (LDL-C) and high-density lipoprotein cholesterol (HDL-C) in the weaned pigs were estimated using porcine commercial assay kits (Nanjing Jiancheng Bioengineering Institute, Nanjing, China), as described before [15]. The contents of triiodothyronine (T3), tetraiodothyronine (T4), insulin, glucagon-like peptide 1 (GLP-1), growth hormone-releasing peptide (GHRP), cholecystokinin (CCK), growth hormone (GH), somatostatin (SS) and leptin in the plasma of the weaned pigs were determined using porcine ELISA kits (Pengguang Biotechnology, Chongqing, China). The liver samples (approximately 100 mg) were added to 900 μL of normal saline, centrifuged at 3500 rpm at 4 °C for 10 min after homogenization and the supernatant was used to measure the contents of glucokinase (GK), lactate dehydrogenase (LDH), pyruvate dehydrogenase (PDH) and glutamate dehydrogenase (GDH) using porcine enzyme-linked immunosorbent assay (ELISA) kits. All procedures were performed according to the manufacturers’ instructions.

#### 4.3.3. Untargeted Metabolomic Analysis

The liver tissues (100 mg per sample) were placed in a 2 mL centrifuge tube and mixed with 1000 µL 75% extract solution (methanol:chloroform = 9:1, *v*/*v*). The mixture was homogenized (at 50 Hz for 60 s, twice), ultrasonicated for 30 min and placed in an ice bath for 30 min. The mixture was then centrifuged at 12,000 rpm and 4 °C for 10 min; then, the supernatant was collected in 2 mL centrifuge tubes. The samples were redissolved in 200 μL of 50% acetonitrile solution prepared with 2-amino-3-(2-chlorophenyl)-propionic acid (4 ppm) for LC-MS analysis. The quality control (QC) sample was prepared by mixing equal volumes of supernatants from all samples.

The analyses were performed using an ACQUITY system (Waters, Milford, MA, USA) with an ACQUITY UPLC^®^ HSS T3 Amide column (2.1 × 150 mm, 1.8 μm) coupled with a Q Exactive HFX mass spectrometer (Orbitrap MS, Thermo Fisher Scientific), as described before [49,50]. The raw data were converted into mzXML files using the MSConvert in ProteoWizard software package and were processed using XCMS in the Ropls software package for feature detection, extraction, alignment and integration. Ropls software was used to analyze the multivariate data, including principal component analysis (PCA), partial least square discriminant analysis (PLS-DA) and orthogonal partial least square discriminant analysis (OPLS-DA). The PCA was applied to obtain the general profiles and clustering of the metabolic data. The OPLS-DA was used for statistical analysis to determine the global metabolic changes among groups, and the metabolites with the variable importance in the projection (VIP) value > 1 and *p*-values < 0.05 were deemed statistically significance. The identified metabolites in the metabolomics were subsequently mapped to pathways in the Kyoto Encyclopedia of Genes and Genomes (KEGG) for biological interpretation of higher-level systemic functions.

#### 4.3.4. Quantitative Real-Time PCR

Total RNA was extracted from the liver using an RNA Quick Extraction kit (Accurate, Changsha, China). The yield and the purity of the RNA was assessed using NanoDrop 1000 (Thermo Fisher Scientific, Waltham, MA, USA). Subsequently, 1 μg of RNA was reverse transcribed to cDNA using an AMW Fist Strand cDNA Synthesis Kit (Applied Biological Materials, Vancouver, BS, Canada) according to the manufacturer’s instructions. A real-time PCR analysis was performed using the SYBR Green method on the CFX Connect Detection System (Bio-Rad, Hercules, CA, USA). PCR amplification was performed in each 20 μL reaction containing 10 μL master mix (Applied Biological Materials, Vancouver, Canada), 0.5 μL forward primer (10 μM), 0.5 μL reverse primer (10 μM), 2 μL cDNA and 7 μL ddH_2_O. The thermal cycling protocol consisted of 3 min at 95 °C followed by 40 cycles of 15 s at 95 °C and 1 min at 60 °C. *GAPDH* was used as a housekeeping gene in this study, and the sequences for the *GAPDH* and target genes are listed in Table 6. The relative mRNA expression of the target genes was calculated for each sample using the 2^−ΔΔCt^ method, as described [51]. 

### 4.4. Statistical Analyses

All experimental data were analyzed using SAS software (version 9.2; SAS Institute Inc., Cary, NC, USA). The data were analyzed using one-way analysis of variance (ANOVA) with Duncan’s multiple range post hoc test. All data are presented as the mean ± standard error of the mean (SEM). Differences were considered statistically significant at *p* < 0.05.

## Figures and Tables

**Table 1:** Effects of LP diet supplemented with SB, MCFAs and n-3 PUFAs on the organ weight and relative weight of the organs of weaned pigs.

Item	Dietary Treatments ^1^					SEM	*p*-Value
	CON	LP	LP + SB	LP + MCFA	LP + PUFA		
Organ Weight (g)							
Liver	391.9	403.1	373.9	396.0	396.0	7.35	0.799
Heart	85.93	87.02	85.67	83.82	83.17	1.23	0.876
Kidney	78.85	75.48	76.85	74.98	78.07	1.77	0.960
Pancreas	22.55	24.42	18.18	23.30	21.88	0.80	0.125
Spleen	33.65	32.12	27.43	36.25	35.90	1.40	0.273
Relative weight of organ (g/kg)							
Liver	23.16 ^b^	28.55 ^a^	27.32 ^a^	29.85 ^a^	29.37 ^a^	0.67	0.005
Heart	5.09 ^b^	6.17 ^a^	6.24 ^a^	6.32 ^a^	6.17 ^a^	0.12	0.001
Kidney	4.67	5.35	5.63	5.64	5.78	0.14	0.078
Pancreas	1.39 ^b^	1.73 ^a^	1.32 ^b^	1.75 ^a^	1.62 ^ab^	0.06	0.014
Spleen	2.00	2.28	2.00	2.73	2.66	0.11	0.052

^1^ CON, 20.4% CP diet; LP, 16.5% CP diet; LP + SB, LP diet supplemented with 0.2% sodium butyrate; LP + MCFA, LP diet supplemented with 0.2% MCFAs; LP + PUFA, LP diet supplemented with 0.2% n-3 PUFAs. ^a, b^ Values within a row with different superscripts differ significantly (*p* < 0.05). Data are presented as mean ± SEM; 6 replicates/treatment (*n* = 6), 1 pig/replicate.

**Table 2:** Effects of LP diet supplemented with SB, MCFAs and n-3 PUFAs on the apparent total tract digestibility of weaned pigs.

Items	Dietary Treatments ^1^					SEM	*p*-Value
	CON	LP	LP + SB	LP + MCFA	LP + PUFA		
Dry matter, %	90.42 ^b^	90.53 ^b^	92.03 ^ab^	93.08 ^a^	91.35 ^ab^	0.28	0.006
Crude protein, %	87.32	85.73	87.42	89.72	86.86	0.47	0.096
Ca, %	73.63	76.14	78.75	83.01	74.52	1.19	0.078
Total P, %	72.72 ^c^	73.54 ^c^	80.00 ^ab^	84.88 ^a^	76.29 ^bc^	1.05	<0.001

^1^ CON, 20.4% CP diet; LP, 16.5% CP diet; LP + SB, LP diet supplemented with 0.2% sodium butyrate; LP + MCFA, LP diet supplemented with 0.2% MCFAs; LP + PUFA, LP diet supplemented with 0.2% n-3 PUFAs. ^a, b, c^ Values within a row with different superscripts differ significantly (*p* < 0.05). Data are presented as the mean ± SEM; 6 replicates/treatment (*n* = 6), 1 pig/replicate.

**Table 3:** Effects of LP diet supplemented with SB, MCFAs and n-3 PUFAs on biochemical parameters in the plasma of weaned pigs.

Items	Dietary Treatments ^1^					SEM	*p*-Value
	CON	LP	LP + SB	LP + MCFA	LP + PUFA		
TP (μg/mL)	42.38	42.77	41.81	42.53	41.20	0.49	0.877
PUN (mmol/L)	1.26	1.52	2.84	1.96	2.18	0.22	0.195
Glucose (mmol/L)	5.90	5.35	5.03	5.79	6.38	0.23	0.419
TC (mmol/L)	2.31 ^b^	2.94 ^a^	2.54 ^b^	2.51 ^b^	2.40 ^b^	0.07	0.029
TG (mol/L)	0.57	0.60	0.51	0.56	0.57	0.03	0.953
LDL-C (mmol/L)	0.21	0.19	0.19	0.21	0.18	0.01	0.818
HDL-C (mmol/L)	1.06	1.22	0.97	1.32	1.26	0.05	0.135

^1^ CON, 20.4% CP diet; LP, 16.5% CP diet; LP + SB, LP diet supplemented with 0.2% sodium butyrate; LP + MCFA, LP diet supplemented with 0.2% MCFAs; LP + PUFA, LP diet supplemented with 0.2% n-3 PUFAs. ^a, b^ Values within a row with different superscripts differ significantly (*p* < 0.05). LDL-C, low-density lipoprotein cholesterol; HDL-C, high-density lipoprotein cholesterol; TG, triglycerides; TC, total cholesterol; PUN, plasma urea nitrogen; total protein, TP. Data are presented as the mean ± SEM; 6 replicates/treatment (*n* = 6), 1 pig/replicate.

**Table 4:** Effects of LP diet supplemented with SB, MCFAs and n-3 PUFAs on hormone levels in the plasma of weaned pigs.

Item	Dietary Treatments ^1^					SEM	*p*-Value
	CON	LP	LP + SB	LP + MCFA	LP + PUFA		
Triiodothyronine (pmol/L)	1.60	1.55	1.59	1.65	1.57	0.01	0.125
Tetraiodothyronine (pmol/L)	5.49	5.96	5.66	5.20	5.54	0.12	0.355
Insulin (mIU/L)	0.90	0.60	0.55	2.34	0.88	0.34	0.451
GLP-1 (ng/mL)	0.65	0.60	0.26	1.80	0.78	0.24	0.310
GHRP (ng/mL)	0.87	0.54	0.51	0.66	2.84	0.36	0.191
CCK (pg/mL)	8.35 ^ab^	0.69 ^b^	3.71 ^b^	2.97 ^b^	11.54 ^a^	1.33	0.045
GH (ng/mL)	0.67	0.53	0.45	2.64	0.71	0.32	0.154
Somatostatin (pg/mL)	5.24	3.48	3.32	14.19	6.87	1.73	0.268
Leptin (ng/mL)	0.45	0.26	0.35	1.25	0.45	0.15	0.211

^1^ CON, 20.4% CP diet; LP, 16.5% CP diet; LP + SB, LP diet supplemented with 0.2% sodium butyrate; LP + MCFA, LP diet supplemented with 0.2% MCFAs; LP + PUFA, LP diet supplemented with 0.2% n-3 PUFAs. ^a, b^ Values within a row with different superscripts differ significantly (*p* < 0.05). GLP-1, glucagon-like peptide 1; GHRP, growth hormone-releasing peptide; CCK, cholecystokinin; GH, growth hormone. Data are presented as the mean ± SEM; 6 replicates/treatment (*n* = 6), 1 pig/replicate.

**Table 5:** The ingredients and nutritional composition of the diets (dry matter basis, %).

Items	Dietary Treatments ^1^				
	CON	LP	LP + SB	LP + MCFA	LP + PUFA
Ingredient					
Corn	61.45	69.37	69.23	69.23	69.23
Soybean meal	12.90	8.30	8.28	8.28	8.28
Puffed soybean	12.14	8.20	8.18	8.18	8.18
Fish meal	4.80	4.70	4.69	4.69	4.69
Soybean oil	1.80	1.90	1.90	1.90	1.90
Whey powder	2.50	2.50	2.50	2.50	2.50
CaHPO_4_	1.10	1.24	1.24	1.24	1.24
Limestone	0.80	0.80	0.80	0.80	0.80
NaCl	0.30	0.30	0.30	0.30	0.30
*L*-Lys.HCl	0.66	0.91	0.91	0.91	0.91
*DL*-methionine	0.25	0.32	0.32	0.32	0.32
*L*-tryptophan	0.05	0.09	0.09	0.09	0.09
*L*-threonine	0.25	0.37	0.37	0.37	0.37
Premix ^a^	1.00	1.00	1.00	1.00	1.00
SB	0.00	0.00	0.20	0.00	0.00
MCFAs	0.00	0.00	0.00	0.20	0.00
n-3 PUFAs	0.00	0.00	0.00	0.00	0.20
Total	100.00	100.00	100.00	100.00	100.00
Nutrient levels ^b^					
DE (MJ/kg)	14.67	14.58	14.59	14.59	14.60
CP	20.45	16.75	16.54	16.68	16.15
Lysine	1.56	1.55	1.55	1.55	1.55
Methionine	0.58	0.61	0.61	0.61	0.61
Tryptophan	0.26	0.26	0.26	0.26	0.26
Threonine	0.96	0.96	0.96	0.96	0.96
Cysteine	0.31	0.27	0.26	0.26	0.26
Valine	0.83	0.70	0.70	0.70	0.70
Leucine	1.60	1.41	1.40	1.40	1.40
Isoleucine	0.72	0.60	0.60	0.60	0.60
Arginase	1.15	0.92	0.92	0.92	0.92
Phenylalanine	0.81	0.68	0.67	0.67	0.67
Tryptophan	0.56	0.49	0.49	0.49	0.49
Histidine	0.45	0.39	0.39	0.39	0.39
Ca	0.80	0.89	0.88	0.87	0.82
Total P	0.69	0.74	0.74.	0.77	0.73

^1^ CON, 20.4% CP diet; LP, 16.5% CP diet; LP + SB, LP diet supplemented with 0.2% sodium butyrate; LP + MCFA, LP diet supplemented with 0.2% MCFAs; LP + PUFA, LP diet supplemented with 0.2% n-3 PUFAs. ^a^ The premix provided following per kg of the diet: VA—15,750 IU; VD3—2450 IU; VE—17.5 IU; VK_3_—1.8 mg; VB_1_—2.1 mg; VB_2_—7.0 mg; VB_6_—0.4 mg; VB_12_—0.028 mg; folic acid—0.4 mg; biotin—0.1 mg; niacinamide—28.0 mg; pantothenic—15.8 mg; Zn (as zinc oxide)—170 mg; Fe (as ferrous sulfate)—140 mg; Mn (as manganese sulfate)—34 mg; Cu (as copper sulfate)—16 mg, I (as calcium iodate)—0.55 mg; Se (as sodium selenite)—0.29 mg. ^b^ DE and amino acids are calculated values based on the nutritional values of the NRC ingredients, and the others were measured.

**Table 6:** Primer parameters for qRT-PCR.

Gene	Primer Sequence (5′→3′)	Accession Number	Product Size (bp)
*CPT1*	F: CCACTATG*ACC*CGGAAGACG	NM_001007191.1	111
	R: TTGAACGCGATGAGGGTGAA		
*ACC*	F: TTCCTATCGGCTATTGACA R: CTTCGCACATAC*ACC*TCC	NM_001114269.1	158
*FASN*	F: CGTGGGCTACAGCATGATAGG R: GAGGAGCAGGCCGTGTCTAT	NM_001099930.1	108
*SREBP1*	F: TCCATCAATGACAAGATCATCGA R: CTGGTTGCTCTGCTGAAGGAA	NM_214157.1	123
*GAPDH*	F: ACATCAAGAAGGTGGTGAAG R: ATTGTCGT*ACC*AGGAAATGAG	NM_001206359.1	178

*CPT1*, carnitine palmitoyl transferase 1; *ACC*, acetyl-CoA carboxylase; *FASN*, fatty acid synthase; *SREBP1*, sterol regulatory element-binding protein 1; *GAPDH*, glyceraldehyde phosphate dehydrogenase.

## Data Availability

The data analyzed during the current study are available from the corresponding author upon reasonable request.
